# Bilingual Language Control in Phonological Encoding: Evidence from Chinese–English Bilinguals

**DOI:** 10.3390/bs16010051

**Published:** 2025-12-27

**Authors:** Renhui Hou, Shifa Chen, Yule Peng

**Affiliations:** Department of English, Ocean University of China, Qingdao 266000, China; hrh@stu.ouc.edu.cn (R.H.); chenshifa99@ouc.edu.cn (S.C.)

**Keywords:** bilingual language control, phonological encoding, Language-Specific Selection Model, retrieval-induced forgetting, bilingual picture–word interference

## Abstract

This study explored language control in phonological encoding during L1 (Chinese) and L2 (English) production via two retrieval-induced forgetting (RIF) experiments and two bilingual picture–word interference (PWI) experiments with Chinese–English bilinguals. RIF results showed that performance on a target language phonological judgement task can be facilitated by prior picture naming in either the target language or a non-target language in both L2 and L1 production. Bilingual PWI results revealed cross-language phonological facilitation effects in L2 and L1 production. Domain-general cognitive control only moderated effects in L2 tasks. Findings confirmed non-selective phonological activation of translation equivalents and cross-language phonologically related words and supported the Language-Specific Selection Model as the primary language control mechanism in phonological encoding, which restricts competition to the target language.

## 1. Introduction

A prevalent assumption in bilingualism research posits that when bilinguals generate words in the target language, they simultaneously activate linguistic representations from the non-target language (Colomé & Miozzo, 2010; Costa & Caramazza, 1999; Guo & Peng, 2006; Hoshino & Kroll, 2008). This parallel lexical activation creates an essential requirement for the language control mechanism, which enables the production of the target language while mitigating potential interference from the non-target language (Abutalebi & Green, 2007, 2008; Green & Abutalebi, 2013). To explain the cognitive mechanism underlying language control, two mutually competing theoretical frameworks have been put forward. The first is the Inhibitory Control Model (Green, 1998), which proposes a global suppression mechanism directed at the non-target language to reduce its interference. The second is the Language-Specific Selection Model (Costa et al., 1999), which argues that language control is achieved through selective attention focused on the linguistic representations of the target language, with no direct inhibitory processes acting on the non-target language.

Most established models of language control operate under the assumption that the primary locus of language control resides at the lemma level (Declerck & Philipp, 2015a). Notably, far less attention has been directed toward investigating the potential language control in phonological encoding. Phonological encoding is “the process by which speakers retrieve phonemic segments for morphemes from memory and use the segments to assemble phonological representations of words to be spoken” (Roelofs & Verhoef, 2006, p. 167). Encoding phonological representations in the target language will proceed without complications if the phonological representations of two languages are entirely discrete, as well as if only the phonological representations of the target language are selectively activated during speech production (Roelofs & Verhoef, 2006). However, there is growing theoretical propositions and empirical evidence of non-selective activation of phonological representations, which may trigger language control in phonological encoding. Exploring language control in phonological encoding is inherently multifaceted, as it involves at least two distinct pathways through which cross-language phonological activation may arise.

### 1.1. Language Control via the Pathway of Phonological Activation of Translation Equivalents

The first pathway hinges on the potential phonological activation of translation equivalents. This stems from the potential interaction between lexical selection and phonological encoding, which may influence whether the phonological representations of target word translation equivalents become activated (and thus whether language control mechanisms are triggered). Two influential models of speech production offer contrasting perspectives on this relationship. According to the Discrete Two-Stage Model (Levelt et al., 1999), lexical selection and phonological encoding function as independent, sequential stages with no temporal overlap. Crucially, this model posits that only the target word identified through the lexical selection stage proceeds to enter the phonological encoding stage, where its phonological form is processed. From this theoretical standpoint, the phonological representations of translation equivalents should remain inactive, eliminating the need for language control to operate at the phonological level in this scenario. In contrast, the Interactive Activation Model (Dell, 1986) argues that lexical selection and phonological encoding overlap temporally (and that the phonological encoding stage permits the activation of multiple phonological representations). This creates the potential for the phonological representations of translation equivalents to be activated alongside those of the target word, which would necessitate the engagement of language control functions to suppress non-target phonological activation and ensure accurate target language production.

A substantial body of research has focused on examining whether the phonological representations of translation equivalents undergo activation during bilingual language production. To address this specific research question, prior investigations have adopted a varied set of methodological paradigms, encompassing picture–word interference (PWI) paradigms (Hermans et al., 1998), cued picture naming tasks (Kroll et al., 2000), phoneme monitoring tasks (Colomé, 2001; Hermans et al., 2011), picture–picture interference paradigms (Colomé & Miozzo, 2010), and color naming tasks (Macizo, 2015). Notably, each of these methodological approaches has yielded robust empirical evidence that substantiates the activation of translation equivalents’ phonological representations in language production.

While existing research has confirmed that the phonological representations of translation equivalents are activated during bilingual language production, the mechanism underlying the avoidance of their interference remains inadequately understood. To address this research gap, Levy et al. (2007) turned to retrieval-induced forgetting (RIF) experiments to investigate the role of inhibition in bilingual language production, using English–Spanish bilinguals as participants. First, the bilinguals completed a picture naming task, where they named objects either in L1 or L2. The number of naming trials was manipulated across four conditions: 0 trials (baseline), 1 trial, 5 trials, or 10 trials. Following this naming phase, the participants completed a final test, which was designed to assess the accessibility of L1 phonological representations, where they were required to generate L1 words that rhymed with provided phonological probes. The core hypothesis was that, if inhibition operates during L2 production, retrieving L2 words would suppress their L1 translation equivalents, leading to lower recall rates for these L1 equivalents in the final test. The results yielded a clear pattern: as the number of L2 naming trials increased, the participants showed lower recall rates for the corresponding L1 translation equivalents. This finding supported the presence of inhibitory control over L1 phonological representations during L2 production. Notably, this inhibition-related finding failed to replicate in subsequent studies by Linck (2008) and Runnqvist and Costa (2012). These non-replication results challenge the proposed role of inhibition in bilingual language production and instead support alternative theoretical frameworks, such as the Feature Suppression Model (Anderson & Spellman, 1995). Given the inconsistency and complexity of these findings, further empirical validation and in-depth exploration are required to clarify the mechanisms governing the control of translation equivalents’ phonological interference in bilinguals. Another gap in the existing literature is that these studies have exclusively focused on bilinguals who use similar-script languages, which possibly limits the generalizability of current conclusions. Consequently, it is methodologically and theoretically necessary to extend such investigations to distinct-script bilinguals, such as Chinese–English bilinguals.

### 1.2. Language Control via the Pathway of Cross-Language Phonological Similarity

The second pathway is driven by cross-language phonological similarity, which can independently induce the non-selective activation of phonological representations. Notably, even if lexical selection and phonological encoding are independent processes, consistent with the Discrete Two-Stage Model, this framework does not rule out the activation of non-target language phonology. Specifically, the activation of non-target language phonological representations is thought to occur in proportion to the degree of overlap or shared features between the phonological systems of the two languages in memory (Roelofs & Verhoef, 2006). Many phonemic segments (e.g., /m/, /t/, /p/, and /k/) only exhibit minor articulatory or acoustic differences across the majority of the world’s languages, suggesting that their core phonological features highly overlap (Ladefoged & Maddieson, 1996). This similarity increases the likelihood that activating a phonemic segment in the target language may trigger the activation of its near-identical counterpart in the non-target language, laying the groundwork for the existence of cross-language phonologically similar words. Importantly, empirical evidence supporting the link between cross-language phonological similarity and non-selective phonological activation has been consistently documented in both language comprehension and language production research (e.g., Duyck et al., 2004; Xu et al., 2021; Zhang et al., 2023; Zhou et al., 2010).

Language similarity at the phonological level possibly plays a role in modulating the extent to which bilingual language control mechanisms are recruited (Boukadi et al., 2015; Rodriguez-Fornells et al., 2006). To examine the role of cross-language phonological similarity, a series of studies have employed the language-switching paradigm, though their findings have proven inconsistent and complex. Declerck and Philipp (2015b) focused on the impact of noncognates with partial phonological overlap on switch costs. Their results demonstrated that the asymmetry of switch costs was modulated by manipulating such noncognates. This effect stemmed from the lingering influence of phonological overlap from the current trial, which carried over to affect processing in the subsequent trial, aligning with the Inhibitory Control Model. Several studies have investigated language switching using the letter naming task. For instance, Zuo et al. (2022) employed Chinese–English bilinguals, while Yue et al. (2025) used trilingual speakers of Chinese, English, and German, both of which observed switch and mixing costs linked to inhibitory control. In contrast, Declerck et al. (2013) adopted a different manipulation by comparing switch costs between words containing language-unspecific phonemes and those containing language-specific phonemes. Their results revealed no significant difference in switch costs between these two categories, suggesting that phonological variations in stimulus words do not exert a universal influence on language-switching effects.

Another body of research has investigated cross-language phonological similarity using the bilingual PWI paradigm, where participants are instructed to name pictures in a target language while ignoring distractor words presented in the non-target language. To investigate the role of cross-language phonological similarity, distractors were manipulated to be either phonologically related or unrelated to the picture names. A typical finding is the cross-language phonological facilitation effects in which participants name pictures faster when paired with phonologically related distractors than with unrelated distractors (e.g., Costa et al., 2003; Costa & Caramazza, 1999; Hermans et al., 1998). This effect has been observed in multiple language pairs, including Spanish–Catalan (Costa et al., 2003), Dutch–English (Hermans et al., 1998), and Spanish–English (Costa & Caramazza, 1999), and it is typically interpreted as evidence that non-target language phonology is activated and indirectly boosts processing of the target language’s phonological representations, supporting the Language-Specific Selection Model. However, research has challenged the consistency of this facilitation effect, revealing that it is not as universally replicable as once assumed. Some studies have reported cross-language phonological interference effects (slower naming with related distractors) rather than facilitation effects (Boukadi et al., 2015; Hoshino & Thierry, 2011). Notably, Hoshino et al. (2021) compared cross-language phonological effects across two distinct bilingual groups: Spanish–English bilinguals (similar-script languages) and Japanese–English bilinguals (distinct-script languages). They observed robust facilitation effects in the Spanish–English group but null effects (no difference between related and unrelated distractors) in the Japanese–English group, leading them to conclude that cross-language script difference may act as a critical moderating factor, reducing or eliminating phonological facilitation when scripts are dissimilar. These conflicting patterns underscore the need for further investigation into the nature of cross-language phonological effects in bilingual PWI experiments. Given that most prior research has focused on similar-script language pairs, it is necessary to extend this line of inquiry to distinct-script bilingual populations, such as Chinese–English bilinguals.

### 1.3. The Current Study

Given these two distinct pathways, namely the phonological activation of translation equivalents and cross-language phonological similarity, any rigorous investigation of language control during the phonological encoding stage should incorporate at least these two perspectives. Accordingly, the present study aims to examine language control in phonological encoding by addressing both pathways. A critical review of the aforementioned literature identifies several gaps in existing research. First, studies centered on distinct-script bilingual populations remain relatively scarce, limiting the generalizability of findings across bilingual groups with divergent writing systems. Second, the majority of prior investigations have focused exclusively on language control during L2 production, thereby overlooking the potential modulating role of language dominance in shaping control processes. Third, few studies have explicitly accounted for the influence of individual differences. To address these gaps, the present study intends to recruit Chinese–English bilinguals as participants. Specifically, this study compared, while also exploring the impact of the participants’ domain-general cognitive control ability (which refers to a set of core cognitive functions, such as attention, problem solving, working memory, and inhibition that monitor and control goal-driven behavioral responses) (Mackie et al., 2013), language control processes during both L2 and L1 production.

Specifically, the present study was designed to address two core research questions, each consisting of two sub-questions. These queries are outlined as follows.

Research Question 1: What are the language control mechanisms potentially elicited by the phonological activation of translation equivalents?

Question 1A: When Chinese–English bilingual speakers produce an English word, are the phonological representations of both the target English word and its Chinese translation equivalent co-activated? If co-activation occurs, what language control mechanism is employed to mitigate the potential interference arising from the phonological representation of the Chinese translation equivalent? How does domain-general cognitive control ability influence this process?

Question 1B. When Chinese–English bilingual speakers produce a Chinese word, are the phonological representations of both the target Chinese word and its English translation equivalent co-activated? If co-activation occurs, what language control mechanism is employed to mitigate the potential interference arising from the phonological representation of the English translation equivalent? How does domain-general cognitive control ability influence this process?

Research Question 2. What are the language control mechanisms potentially elicited by cross-language phonological similarity?

Question 2A. When Chinese–English bilingual speakers produce an English word, are the phonological representations of Chinese words that share phonological similarity with the target English word co-activated? If co-activation occurs, what language control mechanism is employed to mitigate the potential interference arising from these Chinese phonological representations? How does domain-general cognitive control ability influence this process?

Question 2B. When Chinese–English bilingual speakers produce a Chinese word, are the phonological representations of English words that share phonological similarity with the target Chinese word co-activated? If co-activation occurs, what language control mechanism is employed to mitigate the potential interference arising from these English phonological representations? How does domain-general cognitive control ability influence this process?

To address Research Question 1, the present study employed Experiments 1A and 1B, targeting sub-questions 1A and 1B, respectively. Both experiments adopted the RIF paradigm, which is designed to examine potential language control mechanisms during L2 and L1 production.

In Experiment 1A, the participants completed two consecutive tasks: a picture naming task and an L1 (Chinese) phonological judgment task. In the naming phase, the participants were instructed to name pictures either in their L1 or L2, with the number of naming trials per item manipulated across three conditions: naming 0 times, naming 3 times, and naming 6 times. Subsequent to the naming phase, the participants performed a Chinese phonological judgment task, where they determined whether the Chinese names of all pictures presented in the naming task were phonologically related to the provided Chinese phonological cues. Experiment 1B paralleled Experiment 1A but centered on L1 production. It adopted the same two-task structure: a picture naming task and an L2 phonological judgment task.

Three competing predictions guided this experiment, with Experiment 1A used as an illustrative example to elaborate on the rationale. First, if lexical selection and phonological encoding conform to the Discrete Two-Stage Model, L2 picture naming should not influence the accessibility of phonological representations corresponding to L1 translation equivalents. In this case, neither reaction time (RT) nor accuracy rate (ACC) in the phonological judgment task would vary as a function of L2 naming trial frequency. Second, if the two processes align with the Interactive Activation Model and inhibition is deployed to mitigate interference from L1 translation equivalents’ phonological representations, L2 naming would reduce the accessibility of these L1 phonological representations. Consequently, in the phonological judgment task, RTs for cues linked to L2 naming trials would be longer than those linked to trials named 0 times. It is further hypothesized that RTs would increase with more L2 naming times (i.e., naming 6 times yielding longer RTs than naming 3 times), with ACCs exhibiting the opposite pattern. Third, if the two processes follow the Interactive Activation Model but interference avoidance relies on the Language-Specific Selection Model rather than inhibition, L2 naming would not suppress L1 translation equivalents’ phonological representations. Instead, it would enhance their accessibility. Under this scenario, RTs for cues corresponding to L2 naming trials would be shorter than those linked to trials named 0 times, and RTs would decrease as L2 naming trial frequency increases (i.e., naming 6 times resulting in shorter RTs than naming 3 times), with ACCs following the inverse trend. Experiment 1B features parallel predictions, centered on the comparison between L1 naming conditions and the baseline condition.

To address Research Question 2, the present study employed Experiments 2A and 2B, targeting sub-questions 2A and 2B, respectively. Both experiments adopted the bilingual PWI paradigm, which is designed to investigate potential language control mechanisms during L2 and L1 production. In Experiment 2A, the participants were instructed to name pictures in English while ignoring Chinese distractors; in Experiment 2B, the participants named pictures in Chinese while ignoring English distractors. Distractors were categorized into two types: those phonologically related to the target picture names and those phonologically unrelated.

The experimental predictions were derived from two competing theoretical frameworks. If the Inhibitory Control Model governs language control in this context, competition between target and distractor representations would be anticipated. Under this model, phonologically related distractors would act as stronger competitors to target names than phonologically unrelated distractors, ultimately inducing a phonological interference effect. Conversely, if the Language-Specific Selection Model was operative, competition would be exclusively restricted to within-language representations. Since distractors would not engage with the target language selection mechanism, they would not compete with target names, resulting in no significant phonological interference effect.

Experiments 1A and 1B were designed to explore the language control mechanisms potentially elicited by the phonological activation of translation equivalents during L2 and L1 production, respectively. Experiments 2A and 2B aimed to examine language control mechanisms triggered by cross-language phonological similarity during L2 and L1 production, respectively. Collectively, these four experiments sought to comprehensively investigate the language control mechanisms in the phonological encoding stage of bilingual language production.

## 2. Materials and Methods

### 2.1. Experiment 1A: Language Control via Phonological Activation of Translation Equivalents in L2 Production

#### 2.1.1. Participants

Sixty-one Chinese–English bilinguals participated in Experiment 1 (42 female; mean age = 20.28, SD = 1.56). All participants were native speakers of Chinese and English majors enrolled at a university in China, and each had successfully passed the Test for English Majors Grade 4 (TEM-4), which is a standardized proficiency examination specifically designed for undergraduate English majors in China. Prior to the experimental task, all participants completed the Vocabulary Size Test (Nation & Beglar, 2007) to assess their English lexical proficiency. Results from this test indicated a mean vocabulary size of 8016.41 words (SD = 2882.53). According to the criteria established by Nation and Beglar (2007), this score exceeded the typical English vocabulary range of 5000–6000 words for undergraduate students and approached the 9000-word benchmark often observed for doctoral students in English-speaking academic settings. In addition to the Vocabulary Size Test, the participants self-rated their proficiency in L1 and L2 across four linguistic domains: speaking, writing, listening, and reading. This self-assessment was conducted using a 7-point Likert scale (MacIntyre et al., 1997), where a score of 1 corresponded to “very poor” proficiency and 7 to “very proficient” proficiency. As detailed in Table 1, combined results from the Vocabulary Size Test and self-ratings confirmed that the participants exhibited medium-to-high English proficiency. Moreover, all participants were right-handed, had normal or corrected-to-normal visual acuity, and reported no history of linguistic disorders or neurological impairments. Notably, all participants in the present study were reviewed and approved by the Ethics Review Committee of the College of Foreign Languages, Ocean University of China (IRB Number: OUCIRB2023013). All procedures were conducted in accordance with the ethical standards of the committee and the 1964 Declaration of Helsinki and its later amendments. Written informed consent was given by all participants before data collection, and additional consent was provided for the publication of any potentially identifiable information.

To assess the participants’ general-domain cognitive control ability, all participants completed a Flanker task (Eriksen & Eriksen, 1974). In this task, participants were presented with a central target arrow surrounded by distractor arrows, and their task was to indicate the direction of the target arrow while ignoring the distracting stimuli. Trials were categorized into congruent trials, where the direction of the distractor arrows matched that of the target arrow, and incongruent trials, where the distractor arrows pointed in the opposite direction to the target. The task followed a procedure: (a) a fixation cross appeared at the center of the screen and remained visible for 1000 ms; (b) a blank screen was presented for 500 ms; (c) the target arrow and flanking distractors were displayed simultaneously for a maximum of 2000 ms (or until the participant provided a response); and (d) immediately after a response was recorded, the target and distractors disappeared (and the next trial started). General-domain cognitive control ability was operationalized using the Flanker effect calculated as the difference in RTs between the incongruent trials and congruent trials.

#### 2.1.2. Materials

Thirty-six pictures were selected as stimuli for the picture naming task, with these stimuli divided into 6 groups of 6 pictures each: two groups served as baseline conditions (0 naming trials); two were assigned to English naming (one group named 3 times and one named 6 times); and the final two to Chinese naming (following the same 3-trial and 6-trial structure). To ensure the comparability of stimulus properties across groups, an independent sample of 30 participants who matched the formal experiment’s participants in language proficiency completed rating tasks to assess key attributes, which were operationalized as follows: name agreement (10 s picture presentations for immediate name provision); image agreement (5 s word-induced mental imagery followed by 3 s picture presentation, rated on a 5-point scale for word picture match); picture familiarity (8 s presentations rated on a 5-point scale for daily experience familiarity); and visual complexity (8 s presentations rated on a 5-point scale for detail/intricacy). One-way ANOVA confirmed no significant differences across the 6 groups in Chinese name agreement (*F*(1, 5) = 0.63, *p* = 0.68), English name agreement (*F*(1, 5) = 0.81, *p* = 0.55), image agreement with Chinese names (*F*(1, 5) = 0.32, *p* = 0.90), image agreement with English names (*F*(1, 5) = 0.55, *p* = 0.74), picture familiarity (*F*(1, 5) = 0.60, *p* = 0.70), and visual complexity (*F*(1, 5) =1.19, *p* = 0.34). Additional ANOVA verified no group differences in the lexical properties of the pictures’ names: for Chinese names (all one-character words), the number of strokes (*F*(1, 5) = 0.09, *p* = 0.99), frequency (*F*(1, 5) = 0.03, *p* = 0.99), and familiarity (*F*(1, 5) = 0.54, *p* = 0.75); for English names, word length (*F*(1, 5) = 0.66, *p* = 0.65), frequency (*F*(1, 5) = 0.30, *p* = 0.91), and familiarity (*F*(1, 5) = 0.37, *p* = 0.86). The properties of the pictures in Experiment 1A are presented in Table 2.

Given that the phonological cues in the phonological judgement task corresponded to the stimuli used in the picture naming task, the stimuli for the phonological judgement task were grouped to align with the grouping structure of the picture naming task. A total of 48 one-character Chinese words were selected as stimuli for this judgement task: 12 served as filler words, which were phonologically unrelated to the Chinese names of the 36 pictures, and the remaining 36 were phonologically related to the Chinese names of these 36 pictures. These 36 phonologically related words were further divided into six groups, matching the six groups of the pictures in the naming task. To validate the properties of these words, 30 independent participants rated four key attributes using a 5-point scale: word familiarity, phonological relatedness to the target pictures, orthographic relatedness to the pictures, and semantic relatedness to the pictures. Stimulus control was implemented to ensure that the 36 target words met two critical criteria of high phonological relatedness to the pictures’ Chinese names (>4.0) and low orthographic and semantic relatedness to the pictures (<2.0). Furthermore, all Chinese words were selected to carry the fourth tone in order to minimize tonal effects. One-way ANOVA confirmed no significant differences in the lexical properties across the six groups of the target words: word frequency (*F*(1, 5) = 0.04, *p* = 0.99), number of strokes (*F*(1, 5) = 0.68, *p* = 0.64), word familiarity (*F*(1, 5) = 0.81, *p* = 0.55), phonological relatedness to corresponding pictures (*F*(1, 5) = 0.18, *p* = 0.97), orthographic relatedness to corresponding pictures (*F*(1, 5) = 0.35, *p* = 0.88), and semantic relatedness to corresponding pictures (*F*(1, 5) = 0.73, *p* = 0.60). Detailed properties of the phonological cues used in Experiment 1A are presented in Table 3.

Of the 36 pictures included in the picture naming task, only 24 were actually named (i.e., subjected to L1 or L2 naming trials), while the remaining 12 served as baseline pictures (with 0 naming trials). This distinction directly determined the expected responses for the 48 one-character Chinese words in the phonological judgement task: specifically, 24 of these words, which were phonologically related to the Chinese names of the 24 named pictures, required a “related” response indicating a phonological relationship. In contrast, an “unrelated” response was required for two subsets of words, including the 12 filler words phonologically unrelated to all 36 pictures and the 12 words phonologically related to the Chinese names of the 12 baseline pictures. The distribution of stimuli across different experimental conditions and their corresponding expected responses are detailed in Table 4.

#### 2.1.3. Procedure

Participants were individually tested in a sound-attenuated booth, seated approximately 70 cm from a computer monitor. A microphone connected to an electronic voice key was used to capture the vocal responses for the picture naming task, while stimulus presentation and data collection for both tasks were controlled using E-Prime 3.0 software. The experimental session began with a picture familiarization phase, after which participants started a picture naming task. To minimize the language-switching effects during the naming task, stimuli were organized into two blocks separated by naming language. Block order was counterbalanced across participants. Half of the participants completed the English-naming block first, followed by the Chinese-naming block, while the other half completed the blocks in the reverse order. Each picture naming trial followed a standardized sequence: (a) a fixation cross appeared at the screen center and remained for 500 ms; (b) a blank screen was presented for 250 ms; (c) the target picture was displayed for up to 2000 ms, with participants instructed to name the picture aloud using the language specified for the current block; and (d) a random blank of 200–300 ms elapsed before the next trial began. The naming task included 108 total trials (calculated as 2 naming languages × (6 pictures × 3 trials + 6 pictures × 6 trials)), and the participants were given a short break midway through the task.

After completing the picture naming task, participants proceeded to the phonological judgement task. For this task, participants were instructed to judge whether each presented one-character Chinese word (phonological cue) was phonologically related to the Chinese name of any picture they had named in the naming task. Responses were recorded via two designated keys, with the “F” key for “related” and the “J” key for “unrelated”. Key assignments were counterbalanced across participants to control for handedness-related response biases. Each trial was as follows: (a) a fixation cross was displayed for 500 ms; (b) a 250 ms blank screen was presented; (c) the phonological cue (one-character word) appeared for up to 3000 ms, with participants instructed to make a speeded binary judgement; and (d) a random 200–300 ms blank appeared and then the next trial began. RTs and ACC were recorded for each trial.

#### 2.1.4. Data Analysis

Data from the phonological judgement task were analyzed. Responses exceeding 3 SDs and fillers were removed. Statistical analyses were conducted in R (Version 3.2.4) using linear mixed models (LMMs) for RT data and generalized linear mixed models (GLMMs) for ACC. Both models were implemented using the lme4 (Bates et al., 2015) and lmerTest (Kuznetsova et al., 2017) packages. Two sets of models were constructed. The first set incorporated two fixed effects: (1) experimental conditions (naming 0 times vs. English naming vs. Chinese naming) and (2) participants’ general-domain cognitive control ability. Random effects included participants and items. When a significant interaction was detected, simple effect analyses were conducted. The second set focused on separate analyses conducted for English and Chinese naming conditions. For either English naming or Chinese naming, the fixed effects included (1) experimental condition (three levels of naming 0 times, naming 3 times, and naming 6 times) and (2) general-domain cognitive control ability, while the random effects included participants and items. As with the first model set, significant interactions prompted additional simple effect analyses.

### 2.2. Experiment 1B: Language Control via Phonological Activation of Translation Equivalents in L1 Production

#### 2.2.1. Participants

The same participants of Experiment 1A were adopted.

#### 2.2.2. Materials

Thirty-six pictures were selected as stimuli for the picture naming task. One-way ANOVA confirmed no significant differences across the 6 groups in English name agreement (*F*(1, 5) = 0.39, *p* = 0.85), Chinese name agreement (*F*(1, 5) = 0.82, *p* = 0.55), image agreement with English names (*F*(1, 5) = 0.05, *p* = 0.99), image agreement with Chinese names (*F*(1, 5) = 0.42, *p* = 0.83), picture familiarity (*F*(1, 5) = 0.25, *p* = 0.94), and visual complexity (*F*(1, 5) = 0.61, *p* = 0.69). The English names of the pictures were all monosyllabic. Additional ANOVA verified no group differences in the lexical properties of the pictures’ names: for English names, word length (*F*(1, 5) = 0.18, *p* = 0.97), frequency (*F*(1, 5) = 0.17, *p* = 0.97), and familiarity (*F*(1, 5) =1.18, *p* = 0.34); for Chinese names (all one-character words), the number of strokes (*F*(1, 5) = 0.80, *p* = 0.56), frequency (*F*(1, 5) = 0.33, *p* = 0.89), and familiarity (*F*(1, 5) = 0.78, *p* = 0.57). The properties of the pictures in Experiment 1B are presented in Table 5.

A total of 48 monosyllabic English words were selected as stimuli for this judgement task, with 12 words as fillers and 36 words as critical phonological cues. The 36 words were highly phonologically related to the pictures’ English names (>4.0) but not semantically related to the pictures (<2.0). One-way ANOVA confirmed no significant differences in the lexical properties across the six groups of target words: word frequency (*F*(1, 5) = 0.05, *p* = 0.99), length (*F*(1, 5) = 0.26, *p* = 0.93), word familiarity (*F*(1, 5) = 0.17, *p* = 0.97), phonological relatedness to corresponding pictures (*F*(1, 5) = 0.13, *p* = 0.99), and semantic relatedness to corresponding pictures (*F*(1, 5) = 0.60, *p* = 0.70). Detailed properties of the phonological cues used are presented in Table 6.

#### 2.2.3. Procedure

The overall experimental procedure was consistent with that of Experiment 1A.

#### 2.2.4. Data Analysis

The data analysis followed the same rigorous framework as Experiment 1A.

### 2.3. Experiment 2A: Language Control via Cross-Language Phonological Similarity in L2 Production

#### 2.3.1. Participants

Fifty-five Chinese–English bilinguals participated in Experiment 2A (39 female; mean age = 20.36, SD = 1.57). All participants were native speakers of Chinese and English majors enrolled at a university in China, and each had successfully passed the Test for English Majors Grade 4 (TEM-4), which is a standardized proficiency examination specifically designed for undergraduate English majors in China. They showed a mean vocabulary size of 8113.66 words (SD = 2995.13). Table 7 demonstrates their self-rated language proficiency. Combined results from the Vocabulary Size Test and self-ratings confirmed that the participants exhibited medium-to-high English proficiency. Moreover, all participants were right-handed, had normal or corrected-to-normal visual acuity, and reported no history of linguistic disorders or neurological impairments.

#### 2.3.2. Materials

Thirty-six pictures were selected as targets according to the following standards: (a) demonstrating relatively high name agreement (>80%), high image agreement (>4.0 on a 5-point scale), high picture familiarity (>4.0 on a 5-point scale), and low visual complexity (<3.0 on a 5-point scale); (b) possessing monosyllabic English names comprising 3–10 letters, with relatively high word familiarity (>4.0 on a 5-point scale). The properties of the 36 pictures are shown in Table 8.

Two pairs of the 36 one-character Chinese words were selected as distractors. A cohort of 30 assessors matched to the formal experiment’s participants in terms of language proficiency rated the familiarity, phonological relatedness, and semantic relatedness between the distractors and targets using a 5-point scale (1 = very unfamiliar or very unrelated; 5 = very familiar or very related). The two sets of distractors exhibited no significant differences in the number of strokes (*t* (35) = −1.22, *p* = 0.23), frequency (*t* (35) = −0.98, *p* = 0.33), familiarity (*t* (35) = 0.71, *p* = 0.48), and semantic relatedness (*t* (35) = 1.58, *p* = 0.12). The phonologically related distractors showed significantly greater phonological overlap with the picture names compared to the unrelated distractors (*t* (35) = 57.10, *p* < 0.001). All the distractors were one-character words with the fourth tone. As noted in prior phonological research, this tonal pattern is the Mandarin tone most analogous to English’s falling intonation patterns (Chiang, 1979; Xu et al., 2021). For example, for the target, the picture name was “book”, and the phonologically related and unrelated distractors were “布” (/bu/, cloth) and “唱” (/chang/, sing), respectively. The properties of the distractors are demonstrated in Table 9.

#### 2.3.3. Procedure

The participants individually completed the experiment in a sound-attenuated booth, positioned approximately 70 cm from a computer monitor. Vocal responses were captured using a microphone connected to an electronic voice key, while stimulus presentation and data collection were controlled via E-Prime 3.0 software. Prior to the formal task, participants completed a picture familiarization phase. After this phase, participants received task instructions to name each picture as quickly and accurately as possible in English after picture onset, as well as to ignore the distractor words presented alongside the pictures. Each experimental trial followed a standardized sequence: (1) a fixation appeared at the screen center for 500 ms; (2) a blank screen was displayed for 250 ms; (3) the target picture and distractor word were simultaneously presented at the screen center and remained visible for up to 2000 ms; and (4) a variable blank of 200–300 ms elapsed before the next trial began. Each target picture was repeated 3 times across, resulting in a total of 216 trials (36 pictures × 2 distractor conditions × 3 repetitions). The trials were presented in a pseudo-random order to prevent sequential effects, and the participants were given a short rest period after every 72 trials.

#### 2.3.4. Data Analysis

Responses that were incorrect, hesitant, or failed to stop the timer were excluded, as were responses below 100 ms or above 2000 ms, according to the exclusion standard in Roelofs et al. (2016). LMMs were employed to analyze the RT data. The LMMs included two fixed effects: (1) the experimental condition (phonologically related distractors vs. phonologically unrelated distractors), and (2) the participants’ general-domain cognitive control ability. The random effects incorporated participants and items. When a significant interaction was detected, simple effect analyses were conducted.

### 2.4. Experiment 2B: Language Control via Cross-Language Phonological Similarity in L1 Production

#### 2.4.1. Participants

Fifty-seven Chinese–English bilinguals participated in Experiment 2B (40 female; mean age = 20.30, SD = 1.52). All participants were native speakers of Chinese and English majors enrolled at a university in China, and each had successfully passed the Test for English Majors Grade 4 (TEM-4), which is a standardized proficiency examination specifically designed for undergraduate English majors in China. They showed a mean vocabulary size of 8100.02 words (SD = 2932.80). Table 10 demonstrates their self-rated language proficiency. Combined results from the Vocabulary Size Test and self-ratings confirmed that the participants exhibited medium-to-high English proficiency. Moreover, all participants were right-handed, had normal or corrected-to-normal visual acuity, and reported no history of linguistic disorders or neurological impairments.

#### 2.4.2. Materials

Thirty-six pictures were selected as targets. There pictures demonstrated relatively high name agreement (>80%), high image agreement (>4.0 on a 5-point scale), high picture familiarity (>4.0 on a 5-point scale), and low visual complexity (<3.0 on a 5-point scale). Additionally, they possessed one-character Chinese names, with relatively high word familiarity (>4.0 on a 5-point scale). The properties of the 36 pictures are shown in Table 11.

Two pairs of 36 monosyllabic English words were selected as distractors. A panel of 30 assessors matched to the formal experiment’s participants in terms of language proficiency rated the familiarity, phonological relatedness, and semantic relatedness between the distractors and targets using a 5-point scale (1 = very unfamiliar or very unrelated; 5 = very familiar or very related). Two pairs were controlled to show no significant difference in length (t(35) = −1.83, *p* = 0.08), frequency (t(35) = 0.29, *p* = 0.77), familiarity (t(35) = 0.69, *p* = 0.50), and semantic relatedness (t(35) = −1.56, *p* = 0.13). The phonologically related distractors exhibited significantly higher phonological overlap with target picture names compared to the unrelated distractors (t(35) = 42.74, *p* < 0.001). The properties of the distractors are demonstrated in Table 12.

#### 2.4.3. Procedure

The overall experimental procedure was consistent with that of Experiment 2A, albeit with one difference: participants were instructed to name pictures in Chinese and ignore English distractors.

#### 2.4.4. Data Analysis

The data analysis followed the same rigorous framework as Experiment 2A.

## 3. Results

### 3.1. Results of Experiment 1A

Table 13 presents the mean RTs and ACC for all conditions in Experiment 1A.

Table 14a details the outputs of the LMMs for RT data. Compared with the condition of naming 0 times, the Chinese naming condition presented significantly shorter RTs (b = −376.20, SE = 127.51, t = −2.95, *p* = 0.01), and the English naming condition also presented significantly shorter RTs (b = −356.78, SE = 130.81, t = −2.73, *p* = 0.01).

Table 14b details the outputs of the GLMMs for the ACC data. Compared with the condition of naming 0 times, the Chinese naming condition presented higher ACC (b = 1.25, SE = 0.24, t = 5.31, *p* < 0.001), but the English naming condition presented no significant difference (b = 0.21, SE = 0.22, t = 0.94, *p* = 0.35).

Table 15a details the outputs of the LMMs for the RT data in the Chinese naming condition. Naming 3 times resulted in significantly shorter RTs (b = 397.01, SE = 150.65, t = 2.64, *p* = 0.01) relative to the condition of naming 0 times, but it also showed no significant differences (b = 36.38, SE = 164.78, t = 0.22, *p* = 0.83) with the condition of naming 6 times.

Table 15b details the outputs of the GLMMs for the ACC data in Chinese naming condition. Naming 3 times resulted in significantly higher ACC (b = −1.10, SE = 0.29, t = −3.80, *p* < 0.001) relative to the condition of naming 0 times, but it also showed no significant differences (b = 0.32, SE = 0.35, t = 0.90, *p* = 0.37) with the condition of naming 6 times.

Table 16a details the outputs of the LMMs for the RT data in English naming. Naming 3 times similarly led to significantly shorter RTs compared to the condition of naming 0 times (b = 357.97, SE = 153.28, t = 2.34, *p* = 0.02). Consistent with the Chinese naming results, no significant differences (b = 5.06, SE = 170.45, t = 0.03, *p* = 0.98) emerged between the 3-time and 6-time English naming conditions.

Table 16b details the outputs of the GLMMs for the ACC data in English naming. No significant differences were found between naming 3 times and naming 0 times (b = −0.22, SE = 0.27, t = −0.83, *p* = 0.41), as well as between naming 3 times and naming 6 times (b = −0.04, SE = 0.31, t = −0.01, *p* = 0.91).

### 3.2. Results of Experiment 1B

Table 17 presents the mean RTs and ACC for all conditions in Experiment 1B.

Table 18a details the outputs of the LMMs for the RT data. Compared with the condition of naming 0 times, English naming condition presented significantly shorter RTs (b = −684.05, SE = 139.25, t = −4.91, *p* < 0.001), and the Chinese naming condition also presented significantly shorter RTs (b = −376.76, SE = 139.59, t = −2.70, *p* = 0.01).

Table 18b details the outputs of the GLMMs for the ACC data. Compared with the condition of naming 0 times, the English naming condition presented significantly higher ACC (b = 1.07, SE = 0.36, t = 2.95, *p* = 0.003), and the Chinese naming condition also presented significantly higher ACC (b = 0.84, SE = 0.35, t = 2.36, *p* = 0.02).

**Figure 1:**
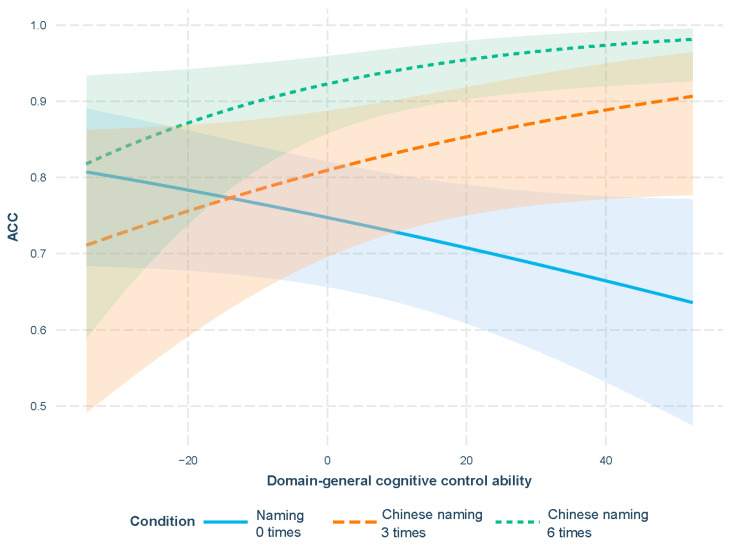
The interaction between the Chinese naming and domain-general cognitive control ability on the ACC in Experiment 1B. Note: The x-axis represents the participants’ domain-general cognitive control ability. The y-axis represents the ACC in Experiment 1B. The shaded areas represent the 95% confidence intervals.

Moreover, a significant interaction between the Chinese naming and domain-general cognitive control ability on ACC was observed (b = 0.03, SE = 0.01, t = 3.27, *p* = 0.001). Based on Figure 1, as the participants’ domain-general Cognitive control ability increased, the ACC in the Chinese naming condition was consistently higher than the ACC in the condition of naming 0 times.

Table 19a details the outputs of the LMMs for the RT data in English naming. Naming 3 times similarly led to significantly shorter RTs compared to the condition of naming 0 times (b = 842.74, SE = 159.70, t = 5.30, *p* < 0.001). No significant differences in the RTs (b = 314.69, SE = 181.96, t = 1.73, *p* = 0.09) emerged between the 3-time and 6-time conditions.

Table 19b details the outputs of the GLMMs for the ACC data in English naming. No significant differences were found between naming 3 times and naming 0 times (b = −0.68, SE = 0.39, t = −1.72, *p* = 0.08), as well as between naming 3 times and naming 6 times (b = 0.81, SE = 0.48, t = 1.67, *p* = 0.10).

Table 20a details the outputs of the LMMs for RT data in the Chinese naming condition. Naming 3 times resulted in significantly shorter RTs compared to the condition of naming 0 times (b = 408.07, SE = 160.59, t = 2.54, *p* = 0.01). When comparing the 3-time and 6-time Chinese naming conditions, no significant difference in the RTs was observed (b = 63.67, SE = 182.95, t = 0.35, *p* = 0.73).

Table 20b details the outputs of the GLMMs for the ACC data in the Chinese naming condition. No significant differences were found between naming 3 times and naming 0 times (b = −0.36, SE = 0.38, t = −0.95, *p* = 0.34). However, the ACC was significantly higher in the 6-time condition (b = 1.03, SE = 0.47, t = 2.22, *p* = 0.03) than the 3-time condition. Additionally, a significant interaction was detected between naming 0 times and the domain-general cognitive control ability on the ACC (b = −0.03, SE = 0.01, t = −2.49, *p* = 0.01), which explained the underlying mechanism of the previously observed interaction between Chinese naming and the domain-general cognitive control ability on the ACC.

### 3.3. Results of Experiment 2A

Table 21 presents the mean RTs and ACC of the different conditions in Experiment 2A. Table 22 shows the results of the LMMs for the RT data. A significant cross-language phonological facilitation effect was obtained (b = 56.57, SE = 3.91, t = 14.47, *p* < 0.001).

**Figure 2:**
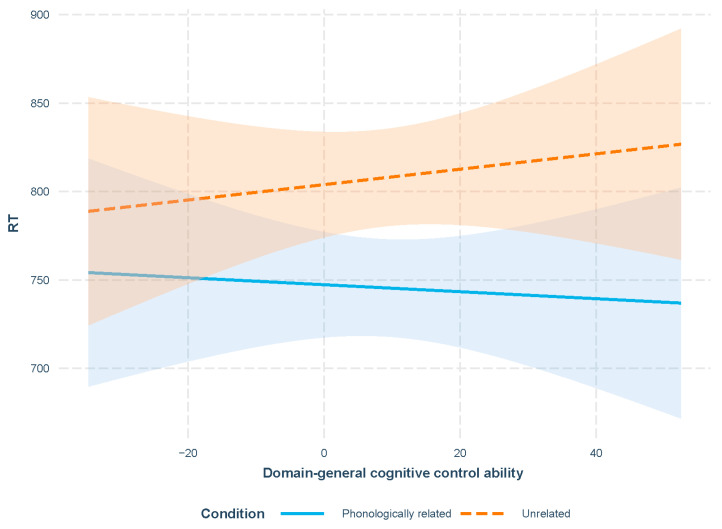
The interaction between the phonological relatedness and domain-general cognitive control ability. Note: The x-axis represents the participants’ domain-general cognitive control ability. The y-axis represents the RTs in Experiment 2A. The shaded areas represent the 95% confidence intervals.

Additionally, an interaction between the phonological relatedness and domain-general cognitive control ability was obtained. According to Figure 2, with an increase in the participants’ domain-general cognitive control ability, a larger cross-language phonological facilitation effect was obtained.

### 3.4. Results of Experiment 2B

Table 23 presents the mean RTs and ACC of the different conditions in Experiment 2B. Table 24 shows the results of the LMMs for the RT data. A significant cross-language phonological facilitation effect was obtained (b = 36.98, SE = 3.94, t = 9.40, *p* < 0.001).

## 4. Discussion

### 4.1. Language Control via Phonological Activation of Translation Equivalents

The results from Experiment 1A demonstrated the influence of the picture naming task on the subsequent performance on the Chinese phonological judgement task. L1 naming significantly facilitated the performance relative to the baseline condition (0 naming trials), in which participants exhibited shorter RTs and higher ACC when judging the phonological relationships between the cues and target picture names after engaging in L1 naming. This finding directly validates the effectiveness of the experiment’s design, as it confirms that L1 naming modulates the accessibility of phonological representations in a manner that translates to improved performance on a downstream phonological processing task. Specifically, the act of naming pictures in L1 increased the activation level of the Chinese phonological representations corresponding to the target pictures, which reduced the cognitive effort required to identify the phonological relationships during the judgement task, thereby leading to superior performance.

A more striking and theoretically informative finding was that L2 naming also facilitated performance on the Chinese phonological judgment task, mirroring the facilitatory effect observed with L1 naming. This result provides compelling evidence that naming pictures in the L2 not only activates the phonological representations of the target English words, but it also enhances the accessibility of phonological representations corresponding to their L1 (Chinese) translation equivalents. Such robust cross-language phonological activation aligns with the converging predictions of the Interactive Activation Model and the Language-Specific Selection Model, offering a coherent mechanistic account of the observed facilitation.

The Interactive Activation Model serves as a foundational framework here, as it posits that not only the target lemma, but also other co-activated lemmas in lexical selection gain access to the phonological encoding stage, enabling the simultaneous activation of multiple phonological representations. Specifically, the model argues that activating a target word’s phonological representation in one language triggers the phonological activation of its translation equivalent. Complementing this, the Language-Specific Selection Model clarifies the critical role of language-specific competition constraints. Notably, the present study extended the original Language-Specific Selection Model to the phonological level. The Language-Specific Selection Model originally proposed that within-language competition occurs at lexical selection, with non-target language lemmas not inhibited but rather excluded from selection. Extending this to the phonological level, non-target language phonological representations are not inhibited but remain activated.

Taken together, these two models synergistically explain the observed facilitation effect in the Chinese phonological judgment task. When participants named pictures in L2, the Interactive Activation Model led to co-activation of the phonological representations of L2 target words and their L1 translation equivalents. Crucially, under the extended Language-Specific Selection Model’s framework, this cross-language activation did not incite between-language competition during phonological encoding. As a result, the activated L1 phonological representations remained functionally accessible and were not subjected to inhibitory suppression. When participants subsequently completed the Chinese phonological judgment task, these pre-activated L1 phonological representations conferred a processing advantage: the prior activation reduced the cognitive effort required to retrieve or verify the phonological relationship between the cues and the L1 translation equivalents, leading to faster RTs and higher ACCs relative to the baseline condition where no prior naming trials had primed the L1 representations.

This pattern of results reinforces the compatibility of the Interactive Activation Model’s non-selective activation account and the Language-Specific Selection Model’s language-specific competition principle. It further highlights that cross-language activation does not inherently lead to interference; instead, the scope of competition determines whether activated non-target representations will hinder or assist subsequent processing. In this case, the absence of cross-language competition allowed the pre-activated L1 phonological representations to serve as a processing scaffold, rather than a competing distraction, thereby enhancing performance on the L1 phonological judgment task.

Further nuanced analysis of the naming time effects revealed an important boundary condition to this facilitatory pattern: significant differences in phonological judgement performance were observed between naming 3 times and naming 0 times, but no significant differences emerged between the naming 3 times and naming 6 times. One plausible explanation for this ceiling effect is that 3 naming trials were sufficient to elevate the activation level of both the target language phonological representations and their translation equivalents to a near-maximal level. Additional naming trials (i.e., increasing from 3 times to 6 times) did not provide further activation gains, as the representations had already reached a threshold where additional exposure did not translate to measurable improvements in downstream task performance.

Experiment 1B yielded results consistent with those of Experiment 1A, providing converging evidence that language control during phonological encoding in L1 production adheres to the combined framework of the Interactive Activation Model and the Language-Specific Selection Model. Specifically, Experiment 1B demonstrated that L1 picture naming coactivated the phonological representations of the corresponding L2 translation equivalents. However, competitive processes during phonological encoding were exclusively restricted to the target language, meaning that the coactivated L2 phonological representations did not compete with L1 target representations for selection.

Beyond replicating the core findings, Experiment 1B demonstrated overall higher ACC than Experiment 1A, which initially appeared counterintuitive given the tasks’ language contexts. Experiment 1B implemented an L2 (English) phonological judgement task, whereas Experiment 1A featured an L1 (Chinese) phonological judgement task. Conventionally, one would anticipate superior performance in L1 tasks, as L1 represented the dominant language for all participants in this study. This unexpected pattern, however, can be unpacked through an analysis of language-specific orthographical–phonological consistency and the strategic deployment of orthographic cues during phonological evaluation. In Experiment 1B, phonological cues were delivered in written form, enabling the participants to utilize orthographic representations as a supportive cue for accessing and evaluating L2 phonological targets. Specifically, the participants could align these orthographic cues with the L2 names of the pictures they had previously named due to English’s inherent properties as an alphabetic language. English exhibits strong mapping between orthographic and phonological outputs. In contrast, Experiment 1A relied on Chinese, a logographic language, where the connection between orthographic and phonological representations is far less systematic. Unlike alphabetic systems, Chinese characters do not encode phonological information through consistent letter–sound correspondences. This weak orthographical–phonological association diminished the utility of orthographic cues for L1 phonological judgement, contributing to the lower ACC observed in Experiment 1A.

In addition, Experiment 1B also identified a novel moderating role of the participants’ domain-general cognitive control ability, in which the participants with higher domain-general cognitive control ability presented a larger facilitation effect on ACC. One possible explanation involved domain-general proactive control. Proactive control refers to “a sustained and anticipatory mode of control that is goal-directed, allowing individuals to actively and optimally configure processing resources prior to the onset of task demands” (Tang et al., 2022, p. 1457). As L2 represents the non-dominant language for all the participants, the L2 phonological judgement task was perceived as more cognitively demanding than the L1 phonological judgement task in Experiment 1A. This perceived difficulty motivated the participants to actively allocate greater cognitive effort to task engagement and goal maintenance before they started L2 task. Therefore, the participants with stronger domain-general cognitive control exhibited superior performance in the L2 task.

The current study’s findings align with those of Linck (2008) and Runnqvist and Costa (2012) as all three studies demonstrated that performance on a target language phonological judgement task can be facilitated by prior picture naming in either the target language or a non-target language. However, the current work extends these prior results by addressing key gaps in their samples and task designs. Linck (2008) conducted four experiments with English–Spanish and Spanish–English bilinguals, investigating both L2 and L1 production effects but focusing exclusively on Indo-European language pairs. Runnqvist and Costa (2012) used Spanish–English and Spanish–Catalan bilinguals spanning multiple L2 proficiency levels but limited their analysis to L2 production. The current study complemented these efforts by extending the facilitatory effect to distinct-script bilinguals and examining both L1 and L2 production contexts, thereby enhancing the generalizability of the facilitation effects. Beyond empirical extensions, the current study also offers a unique theoretical account that diverges from the explanations proposed by Linck (2008) and Runnqvist and Costa (2012). Linck (2008) advanced two tentative interpretations for their facilitatory effects: either (1) language production does not involve inhibitory mechanisms, or (2) inhibition exists but remains undetected due to task-specific factors, such as naming repetition counts and participant L2 proficiency. This account lacked clarity regarding the role of inhibition in phonological encoding. Runnqvist and Costa (2012), by contrast, framed their results as support for the Feature Suppression Model, which posits that inhibition and activation co-occur during memory retrieval: semantically related representations share some features, which drive facilitation and differ in others, triggering inhibition, with the final outcome reflecting a trade-off between these two processes. This model predicts that facilitation should dominate when retrieving words in Language A after practicing them in Language B, which is consistent with their findings. However, Runnqvist and Costa (2012) acknowledged that this account “remains silent about how the bilingual speaker manages to restrict language production to only one language” (p. 10). In response to these accounts, the current study proposes an alternative framework that integrates the Interactive Activation Model and the Language-Specific Selection Model. Notably, it is acknowledged that bilingual language production is a complex process requiring the coordination of multiple cognitive mechanisms. Future research is needed to disentangle the contributions of distinct cognitive mechanisms and to explore their dynamic interactions during bilingual language production.

A further critical point of comparison is the divergence between the current study and Levy et al. (2007). Levy et al. (2007) observed interference in L1 phonological judgement after repeated L2 naming, whereas the current study found facilitation. To explore the potential sources of this discrepancy, three key task and sample differences were examined. First, the number of naming repetitions varied. Levy et al. (2007) implemented 0, 1, 5, or 10 naming times, while the current study used 0, 3, or 6 times. Although one might hypothesize that more repetitions could elicit inhibition, Runnqvist and Costa (2012) also used 0, 1, 5, or 10 times and still observed facilitation, ruling out repetition count as a sole driver of interference. Second, the participants’ L2 proficiency differed. Levy et al. (2007) focused on low-proficiency bilinguals, while the current study included medium-to-high proficiency participants. Again, this cannot fully explain the discrepancy, as Runnqvist and Costa (2012) included bilinguals across low, medium, and high proficiency levels and consistently found facilitation. Third, Levy et al. (2007) relied solely on ACC, while the current study incorporated both RTs and the ACC as dependent variables. As noted in prior research (Veling & van Knippenberg, 2004), RTs and ACC together provide a more sensitive index of underlying cognitive processes than ACC alone. Whereas the ACC captures the accuracy of performance, RTs reflect the efficiency of cognitive processing, enabling detection of subtle effects that may not manifest as errors but instead as delays in resolving interference. Collectively, these analyses suggest that the current study not observing interference effects is not attributable to these task or sample factors but instead provides evidence that the phonological representations of translation equivalents are not inhibited during bilingual production. Nevertheless, additional research is needed to confirm whether inhibition is truly unnecessary for phonological control in bilinguals or simply remains undetected under certain experimental conditions.

### 4.2. Language Control via Cross-Language Phonological Similarity

Experiments 2A and 2B both yielded consistent cross-language phonological facilitation effects, in which the participants named pictures significantly faster when presented with cross-language phonologically related distractors than with unrelated distractors. These findings provide empirical support for the Language-Specific Selection Model. Since the original Language-Specific Selection Model was originally developed to account for processes at the lexical selection stage, its core principles should be extended to phonological encoding. A refined theoretical account rooted in Language-Specific Selection Model’s core principles was proposed to illustrate the language control mechanism underlying phonological encoding. Specifically, when a bilingual speaker engages in the phonological encoding of a target word, the activation of the target’s phonological representation triggers the activation of phonologically related representations in the non-target language. Critically, however, phonological selection is restricted to the target language, suggesting that phonological representations in non-target language do not compete with target representations. The facilitation effects become transparent when viewed through this revised Language-Specific Selection Model lens. In the two experiments, participants were presented with phonologically similar distractors in the non-target language. These visual distractors amplified the activation level of their phonological representations. Importantly, because the phonological selection was language-specific, the activated phonological representations in the non-target language did not compete with the target phonological representation. Instead, the phonological similarity between the two enhanced activation transmission, accelerating the encoding of the target’s phonological representation. This facilitation effect was particularly pronounced in the current study due to the high degree of phonemic overlap between the picture names and distractors across languages. For instance, the L1 Chinese character “币” (coin) has a phonological structure of /b/ + /ɪ/, while its L2 English distractor “bee” consists of /b/ + /iː/, which is a near-perfect phonemic correspondence in terms of consonant and vowel quality and syllabic structure. Such close cross-language alignment maximized the activation spread between related representations, amplifying the observed facilitation effect. Consequently, activated phonological representations in the non-target language functioned as a source of activation reinforcement rather than competitors for target representations.

In addition to the facilitation effects, Experiment 2A revealed a significant interaction between cross-language phonological relatedness and domain-general cognitive control ability. Specifically, as the participants’ domain-general cognitive control ability increased, the magnitude of the cross-language facilitation effect in L2 production became significantly larger. Notably, this interaction was absent in Experiment 2B, which is an L1 production task. As hypothesized in prior analyses of Experiment 1B, the interaction likely reflects the involvement of proactive control. For the participants, L2 functions as the non-dominant language, meaning L2 production tasks are perceived as more cognitively demanding than L1 production tasks. This heightened perceived difficulty made participants proactively allocate greater cognitive effort to maintaining the task goal in L2 tasks, resulting in a detectable influence of domain-general cognitive control ability in L2 production but not in L1 production.

The current findings align with prior studies (Costa et al., 2003; Costa & Caramazza, 1999; Hermans et al., 1998) while diverging from others (Boukadi et al., 2015; Hoshino & Thierry, 2011; Hoshino et al., 2021). Hoshino and Thierry (2011) attributed their failure to observe facilitation to the use of repeated picture names as distractors. Boukadi et al. (2015) documented interference effects in Tunisian Arabic–French bilinguals, proposing that phonological dissimilarity between the two languages drove this outcome. Specifically, they argued that phonemes perceived as closely related yet distinct enough to trigger competition between lexical representations, resulting in interference. They also hypothesized that, with the increase in L2 proficiency, such an interference effect should be decreased or even eliminated. Their account centers on phonological dissimilarity as a critical moderator, and greater sensitivity to such differences enables more effective language differentiation, thereby mitigating non-target language interference. Inspired by Boukadi et al. (2015), the present study proposes another potential explanation. While the Language-Specific Selection Model, with the prerequisite that phonological representations have distinct language tags, can account for the facilitation effect observed, we can also consider a scenario where this prerequisite is given up. For the participants, cross-language phonemes with high similarity may lack clear language tagging. When phonemic overlap is substantial yet distinct and not sufficient to induce competition, then facilitation arises. An extreme situation was that the two similar phonemes were totally undistinguishable for our participants. This alternative explanation is consistent with the Speech Learning Model (Flege, 1995). This model posits that L2 sounds resembling L1 phonemes may fail to be perceptually discriminated by late bilinguals, leading to merged phonological representations. Given the sample of late medium–high proficiency bilinguals in the present study, such merging is plausible, potentially explaining why similar phonemes across languages amplified, rather than disrupted, target processing.

Hoshino et al. (2021) observed facilitation in Spanish–English bilinguals but not in Japanese–English bilinguals, attributing this contrast to script specificity. Distinct writing systems act as early language cues, directing attention to the target language. This aligns with the principle of nonselective activation with language-specific selection mechanisms. Notably, their framework also assumes competition is restricted to the target language, and the non-target language does not exert influence on the target language, corresponding to the null effects. However, this cannot fully account for the facilitation effects in the present study. The present study posits that facilitation can be attributed to an additional process in which the activation of distractor phonemes exerts a facilitatory effect on their cross-language analogous phonemes, which form the targets. This process parallels the explanation provided by the Language-Specific Selection model for the robust translation facilitation observed in the bilingual PWI paradigm, wherein target words enhance the activation of their translation equivalents. The present study draws a direct analogy between the lexical-level relationship of words and their translations and the phonological-level association of phonemes and their cross-language counterparts. Such a process possibly also emerges in Hoshino et al. (2021), yet the contrast between their null effects and the current facilitation findings is likely rooted in stimulus design. Hoshino et al.’s (2021) materials featured partial phonological overlap (e.g., English “envelope” /ˈenvəloʊp/ and Japanese “煙突” /eNtotu/ [chimney]), whereas our stimuli paired monosyllabic Chinese characters with English words (e.g., “币” /bɪ/ and “bee” /biː/) exhibiting extensive vowel and consonant overlap. This greater phonemic correspondence may have amplified facilitatory activation in the present study.

### 4.3. Language Control in Phonological Encoding

Synthesizing all experimental results, the present study concludes two core findings in both L2 and L1 production: (1) non-selective phonological activation occurs during phonological encoding, and (2) the language control mechanism governing phonological encoding aligns with the Language-Specific Selection Model, which restricts competition to the target language and thereby prevents interference from the non-target language. Non-selective phonological activation is triggered via at least two distinct pathways. The first pathway stems from the interactive nature of lexical selection and phonological encoding, as described by the Interactive Activation Model. This model posits that there exists multiple phonological activation in phonological encoding, providing the possibility that the phonological representations of the target word and its translation equivalent are both activated. The second pathway driving non-selective activation is cross-language phonological similarity. This occurs because phonological representations are not entirely language-isolated; overlapping phonemic features create activation links between cross-language counterparts. For both pathways of non-selective activation, the Language-Specific Selection Model functions as the primary language control mechanism during phonological encoding. Critical to the Language-Specific Selection Model is its restriction of competitive interactions to the target language: phonological representations from the non-target language do not compete with target representations for selection. Notably, an alternative account for language control on activation driven by cross-language phonological similarity is that phonemes may bear fuzzy language tags. In this scenario, phonemic overlap is substantial but distinct not sufficient to trigger competition, presenting a language-specific selection manner.

Another finding is the moderating role of domain-general cognitive control in L2 phonological judgement tasks and L2 PWI tasks, which resonates with the Dual Mechanisms of Control framework (Braver, 2012; Braver et al., 2007), and it also partially supports the Adaptive Control Hypothesis (Green & Abutalebi, 2013). The Dual Mechanisms of Control framework distinguishes between two qualitatively distinct cognitive control mechanisms, including proactive control and reactive control. Proactive control is a preparatory mechanism that involves sustained goal maintenance and anticipatory suppression of potential interference, while reactive control operates in a “just-in-time” manner, resolving interference only after it occurs. In the current study, participants perceived L2 tasks as more cognitively demanding than L1 tasks. This heightened perceived difficulty motivated the deployment of proactive control to sustain task goals. Consequently, the moderating effect of domain-general cognitive control was only detectable and measurable in L2 tasks. The Adaptive Control Hypothesis, which is a revised iteration of the Inhibitory Control model, extends reactive control accounts of bilingual language selection by incorporating proactive control, recognizing that bilinguals adapt to diverse interactional contexts that demand flexible language management (Green & Abutalebi, 2013). Specifically, the Adaptive Control Hypothesis argues that bilingual language control is not exclusively reactive; proactive control is critical for adapting to dynamic task demands. While the current study found no evidence for reactive control, it strongly supports the Adaptive Control Hypothesis’s emphasis on proactive control in L2 processing. This partial alignment with the Adaptive Control Hypothesis highlights that bilingual language control is context-dependent.

## 5. Conclusions

The present study investigated language control during phonological encoding in L1 and L2 production via two RIF experiments and two bilingual PWI experiments. The two RIF experiments showed that prior picture naming, regardless of in-target or non-target language, facilitated subsequent target language phonological judgement. The two bilingual PWI experiments confirmed robust cross-language phonological facilitation. Collectively, results support two core conclusions: (1) non-selective phonological activation is fundamental to bilingual phonological encoding; and (2) language control in phonological encoding aligns with the Language-Specific Selection Model, which restricts competition to the target language, preventing non-target interference.

The present findings substantially advance bilingual language production theories by clarifying the core mechanisms of phonological encoding and language control, most notably by extending the scope of language control research from lexical selection to the understudied domain of phonological encoding. Crucially, this study validated that cross-language activation operates at the phonological level, while also refining the Language-Specific Selection Model by extending its core principles to phonological processing. This dual contribution resolves prior ambiguities about whether language control mechanisms apply beyond lexical selection, confirming that competition is restricted to the target language during phonological encoding. Beyond theoretical advancements, the findings offer actionable insights for bilingual education and L2 acquisition. Specifically, the findings highlight that L1 exerts a transfer effect on L2 phonological processing—an effect that can be harnessed rather than avoided. To optimize learning efficiency, instruction should leverage cross-language phonological overlaps, with a critical prerequisite: explicitly distinguishing both the similarities and dissimilarities between L1 and L2 phonological systems. This approach allows learners to capitalize on facilitatory cross-language activation while minimizing potential confusion, turning L1 phonological knowledge into a scaffold for L2 acquisition.

Naturally, the present study is not without limitations. Notably, it has not fully accounted for the potential modulatory effects of additional key variables, with L2 proficiency and language similarity being prominent examples. Furthermore, the integration of neuroimaging techniques, such as ERPs and fMRI, into future experimental designs would yield valuable insights. Such approaches would help clarify both the temporal dynamics of bilingual language production and their underlying neural substrates, thereby advancing a more comprehensive understanding of the complex cognitive processes.

## Figures and Tables

**Table 1:** The participants’ self-rated Chinese and English proficiency in Experiment 1A.

Skills	Chinese Proficiency		English Proficiency	
	Mean	*SD*	Mean	*SD*
Listening	5.77	0.96	4.30	1.07
Speaking	5.36	1.10	4.10	1.11
Reading	5.75	1.06	4.98	1.09
Writing	4.85	1.05	4.31	1.07
Total	5.43	1.04	4.42	1.08

**Table 2:** The properties of the pictures in Experiment 1A.

Variables	Mean	*SD*
Name agreement (%)		
Chinese name agreement	97.10	0.05
English name agreement	89.60	0.10
Image agreement		
With Chinese name	4.81	0.09
With English name	4.78	0.12
Picture familiarity	4.79	0.12
Visual complexity	1.97	0.30
Chinese names		
The number of strokes	8.42	3.75
Frequency	268.29	269.23
Familiarity	4.89	0.07
English names		
Length	4.72	1.60
Frequency	76.35	98.09
Familiarity	4.76	0.25

**Table 3:** The properties of the phonological cues in Experiment 1A.

Variables	Mean	*SD*
Frequency	201.87	134.10
The number of strokes	8.22	2.32
Familiarity	4.84	0.08
Phonological relatedness with corresponding pictures	4.11	0.08
Orthographic relatedness with corresponding pictures	1.28	0.24
Semantic relatedness with corresponding pictures	1.29	0.19

**Table 4:** The experimental conditions and expected responses.

Conditions	Picture Names	Cues	Expected Responses
English naming 3 times	Fire	货	Related
English naming 6 times	Drum	故	Related
Chinese naming 3 times	狗	购	Related
Chinese naming 6 times	圆	院	Related
Naming 0 times	Cat/猫	冒	Unrelated

Note: 货 (goods); 故 (old); 狗 (dog); 购 (buy); 圆 (circle); 院 (yard); and 冒 (emit).

**Table 5:** The properties of the pictures in Experiment 1B.

Variables	Mean	*SD*
Name agreement (%)		
English name agreement	90.40	0.09
Chinese name agreement	96.00	0.07
Image agreement		
With English name	4.73	0.14
With Chinese name	4.77	0.13
Familiarity	4.77	0.17
Visual Complexity	1.87	0.23
English names		
Length	4.11	0.75
Frequency	79.51	79.75
Familiarity	4.77	0.18
Chinese names		
The number of strokes	8.83	3.57
Frequency	228.86	208.64
Familiarity	4.89	0.06

**Table 6:** The properties of the phonological cues in Experiment 1B.

Variables	Mean	*SD*
Frequency	161.82	221.12
Length	4.00	0.68
Familiarity	4.73	0.17
Phonological relatedness with corresponding pictures	3.84	0.12
Semantic relatedness with corresponding pictures	1.33	0.12

**Table 7:** The participants’ self-rated Chinese and English proficiency in Experiment 2A.

Skills	Chinese Proficiency		English Proficiency	
	Mean	*SD*	Mean	*SD*
Listening	5.71	0.98	4.25	1.08
Speaking	5.36	1.06	4.07	1.05
Reading	5.67	1.06	4.98	1.11
Writing	4.80	1.01	4.35	1.04
Total	5.39	1.02	4.41	1.07

**Table 8:** The properties of the 36 pictures in Experiment 2A.

Variables	Mean	*SD*
Name agreement (%)	86	0.09
Image agreement	4.73	0.15
Familiarity	4.73	0.19
Visual complexity	1.86	0.24
English names		
Length	4.06	0.66
Frequency	62.99	79.86
Familiarity	4.71	0.21

**Table 9:** The properties of the distractors in Experiment 2A.

Variable	Related		Unrelated	
	Mean	*SD*	Mean	*SD*
The number of strokes	8.61	3.23	9.39	2.25
Frequency	172.13	166.59	172.53	167.05
Familiarity	4.82	0.09	4.81	0.07
Phonological relatedness	3.81	0.28	1.62	0.22
Semantic relatedness	1.25	0.08	1.22	0.12

**Table 10:** The participants’ self-rated Chinese and English proficiency in Experiment 2B.

Skills	Chinese Proficiency		English Proficiency	
	Mean	*SD*	Mean	*SD*
Listening	5.81	0.91	4.37	0.99
Speaking	5.33	1.09	4.16	1.08
Reading	5.75	1.06	5.02	1.08
Writing	4.93	1.02	4.37	1.06
Total	5.46	1.02	4.48	1.05

**Table 11:** The properties of the 36 pictures in Experiment 2B.

Variables	Mean	*SD*
Name agreement (%)	96	0.06
Image agreement	4.80	0.11
Familiarity	4.78	0.11
Visual complexity	1.86	0.31
Chinese names		
The number of strokes	8.94	3.55
Frequency	282.57	527.27
Familiarity	4.86	0.06

**Table 12:** The properties of the distractors in Experiment 2B.

Variable	Related		Unrelated	
	Mean	*SD*	Mean	*SD*
Length	4.11	0.57	4.36	0.64
Frequency	69.40	96.58	69.34	96.41
Familiarity	4.67	0.28	4.63	0.24
Phonological relatedness	3.79	0.24	1.63	0.16
Semantic relatedness	1.17	0.07	1.18	0.05

**Table 13:** The mean RT and ACC in Experiment 1A.

Conditions	Mean RT (ms)	ACC (%)
Chinese naming 3 times	2038 (950)	84 (37)
Chinese naming 6 times	2091 (927)	88 (33)
Naming 0 times	2490 (956)	65 (48)
English naming 3 times	2150 (973)	70 (47)
English naming 6 times	2132 (875)	68 (47)

**Table 14:** (**a**) The LMMs of the RT data in Experiment 1A. (**b**) The GLMMs of the ACC data in Experiment 1A.

(**a**)				
Fixed Effects	b	*SE*	t	*p*
Chinese naming	−376.20	127.51	−2.95	0.01
English naming	−356.78	130.81	−2.73	0.01
Cognitive control ability	−5.42	4.36	−1.24	0.21
Chinese naming × Cognitive control ability	1.42	2.96	0.48	0.63
English naming × Cognitive control ability	−0.11	3.15	−0.03	0.97
(**b**)				
Fixed Effects	b	*SE*	Z	*p*
Chinese naming	1.25	0.24	5.31	<0.001
English naming	0.21	0.22	0.94	0.35
Cognitive control ability	−0.003	0.01	−0.54	0.59
Chinese naming × Cognitive control ability	−0.0003	0.01	−0.05	0.96
English naming × Cognitive control ability	0.001	0.007	0.21	0.84

Baseline: Naming 0 times.

**Table 15:** (**a**) The LMMs of the RT data in Chinese naming in Experiment 1A. (**b**) The GLMMs of the ACC data in Chinese naming in Experiment 1A.

(**a**)				
Fixed Effects	b	*SE*	t	*p*
Naming 0 times	397.01	150.65	2.64	0.01
Naming 6 times	36.38	164.78	0.22	0.83
Cognitive control ability	−3.05	4.62	−0.66	0.51
Naming 0 times × Cognitive control ability	−2.36	3.61	−0.66	0.51
Naming 6 times × Cognitive control ability	−1.74	3.81	−0.46	0.65
(**b**)				
Fixed Effects	b	*SE*	Z	*p*
Naming 0 time	−1.10	0.29	−3.80	<0.001
Naming 6 times	0.32	0.35	0.90	0.37
Cognitive control ability	−0.01	0.01	−0.58	0.56
Naming 0 time × Cognitive control ability	0.002	0.01	0.25	0.81
Naming 6 times × Cognitive control ability	0.004	0.01	0.36	0.72

Baseline: Chinese naming 3 times.

**Table 16:** (**a**) The LMMs of the RT data in English naming in Experiment 1A. (**b**) The GLMMs of the ACC data in English naming in Experiment 1A.

(**a**)				
Fixed Effects	b	*SE*	t	*p*
Naming 0 times	357.97	153.28	2.34	0.02
Naming 6 times	5.06	170.45	0.03	0.98
Cognitive control ability	−6.65	4.78	−1.39	0.17
Naming 0 times × Cognitive control ability	1.23	3.82	0.32	0.75
Naming 6 times × Cognitive control ability	2.29	4.37	0.52	0.60
(**b**)				
Fixed Effects	b	*SE*	Z	*p*
Naming 0 times	−0.22	0.27	−0.83	0.41
Naming 6 times	−0.04	0.31	−0.01	0.91
Cognitive control ability	−0.002	0.01	−0.21	0.83
Naming 0 times × Cognitive control ability	−0.001	0.01	−0.14	0.89
Naming 6 times × Cognitive control ability	0.0004	0.01	0.04	0.96

Baseline: English naming 3 times.

**Table 17:** The mean RT and ACC in Experiment 1B.

Condition	Mean RT (ms)	ACC (%)
English naming 3 times	1769 (933)	81 (39)
English naming 6 times	2044 (1184)	91 (29)
Naming 0 times	2602 (1009)	71 (46)
Chinese naming 3 times	2211 (1069)	81 (39)
Chinese naming 6 times	2280 (1175)	92 (27)

**Table 18:** (**a**) The LMMs of the RT data in Experiment 1B. (**b**) The GLMMs of the ACC data in Experiment 1B.

(**a**)				
Fixed Effects	b	*SE*	t	*p*
English naming	−684.05	139.25	−4.91	<0.001
Chinese naming	−376.76	139.59	−2.70	0.01
Cognitive control ability	1.40	4.77	0.29	0.77
English naming × Cognitive control ability	−3.26	3.45	−0.95	0.34
Chinese naming × Cognitive control ability	−0.04	3.43	−0.01	0.99
(**b**)				
Fixed Effects	b	*SE*	Z	*p*
English naming	1.07	0.36	2.95	0.003
Chinese naming	0.84	0.35	2.36	0.02
Cognitive control ability	−0.01	0.01	−1.71	0.08
English naming × Cognitive control ability	−0.004	0.01	−0.46	0.64
Chinese naming × Cognitive control ability	0.03	0.01	3.27	0.001

Baseline: Naming 0 times.

**Table 19:** (**a**) The LMMs of the RT data in English naming in Experiment 1B. (**b**) The GLMMs of the ACC data in English naming in Experiment 1B.

(**a**)				
Fixed Effects	b	*SE*	t	*p*
Naming 0 times	842.74	159.70	5.30	<0.001
Naming 6 times	314.69	181.96	1.73	0.09
Cognitive control ability	1.37	5.26	0.26	0.80
Naming 0 times × Cognitive control ability	0.03	4.28	0.01	0.99
Naming 6 times × Cognitive control ability	−6.08	4.71	−1.30	0.20
(**b**)				
Fixed Effects	b	*SE*	Z	*p*
Naming 0 times	−0.68	0.39	−1.72	0.08
Naming 6 times	0.81	0.48	1.67	0.10
Cognitive control ability	−0.02	0.01	−1.75	0.08
Naming 0 times × Cognitive control ability	0.01	0.01	0.56	0.57
Naming 6 times × Cognitive control ability	0.004	0.01	0.29	0.77

Baseline: English naming 3 times.

**Table 20:** (**a**) The LMMs of the RT data in Chinese naming in Experiment 1B. (**b**) The GLMMs of the ACC data in Chinese naming in Experiment 1B.

(**a**)				
Fixed Effects	b	*SE*	t	*p*
Naming 0 times	408.07	160.59	2.54	0.01
Naming 6 times	63.67	182.95	0.35	0.73
Cognitive control ability	0.57	5.22	0.11	0.91
Naming 0 times × Cognitive control ability	0.83	4.22	0.20	0.84
Naming 6 times × Cognitive control ability	1.52	4.64	0.33	0.74
(**b**)				
Fixed Effects	b	*SE*	Z	*p*
Naming 0 times	−0.36	0.38	−0.95	0.34
Naming 6 times	1.03	0.47	2.22	0.03
Cognitive control ability	0.02	0.01	1.76	0.08
Naming 0 times × Cognitive control ability	−0.03	0.01	−2.49	0.01
Naming 6 times × Cognitive control ability	0.01	0.02	0.82	0.41

Baseline: Chinese naming 3 times.

**Table 21:** The mean RT and ACC in Experiment 2A.

Conditions	Mean RT (ms)	ACC (%)
Phonological related	745 (194)	98 (14)
Phonological unrelated	807 (220)	98 (15)

**Table 22:** The LMMs of the RT data in Experiment 2A.

Fixed Effects	b	*SE*	t	*p*
Phonological relatedness	56.57	3.91	14.47	<0.001
Cognitive control ability	−0.20	0.69	−0.29	0.78
Relatedness × Cognitive control ability	0.63	0.22	2.89	0.004

Baseline: phonological related.

**Table 23:** The mean RTs and ACC in Experiment 2B.

Condition	Mean RT (ms)	ACC (%)
Phonological related	743 (223)	98 (14)
Phonological unrelated	780 (233)	98 (15)

**Table 24:** The LMMs of the RT data in Experiment 2B.

Fixed Effects	b	*SE*	t	*p*
Phonological relatedness	36.98	3.94	9.40	<0.001
Cognitive control ability	−1.01	0.88	−1.15	0.26
Relatedness × Cognitive control ability	0.02	0.21	0.07	0.94

## Data Availability

The data presented in this study are openly available in OSF at https://osf.io/d46yj (accessed on 12 October 2025).
